# XRN1 Stalling in the 5’ UTR of Hepatitis C Virus and Bovine Viral Diarrhea Virus Is Associated with Dysregulated Host mRNA Stability

**DOI:** 10.1371/journal.ppat.1004708

**Published:** 2015-03-06

**Authors:** Stephanie L. Moon, Jeffrey G. Blackinton, John R. Anderson, Mary K. Dozier, Benjamin J. T. Dodd, Jack D. Keene, Carol J. Wilusz, Shelton S. Bradrick, Jeffrey Wilusz

**Affiliations:** 1 Department of Microbiology, Immunology and Pathology, Colorado State University, Fort Collins, Colorado, United States of America; 2 Department of Molecular Genetics and Microbiology, Duke University Medical Center, Durham, North Carolina, United States of America; University of California, San Diego, UNITED STATES

## Abstract

We demonstrate that both Hepatitis C virus (HCV) and Bovine Viral Diarrhea virus (BVDV) contain regions in their 5’ UTRs that stall and repress the enzymatic activity of the cellular 5’-3’ exoribonuclease XRN1, resulting in dramatic changes in the stability of cellular mRNAs. We used biochemical assays, virus infections, and transfection of the HCV and BVDV 5’ untranslated regions in the absence of other viral gene products to directly demonstrate the existence and mechanism of this novel host-virus interaction. In the context of HCV infection, we observed globally increased stability of mRNAs resulting in significant increases in abundance of normally short-lived mRNAs encoding a variety of relevant oncogenes and angiogenesis factors. These findings suggest that non-coding regions from multiple genera of the *Flaviviridae* interfere with XRN1 and impact post-transcriptional processes, causing global dysregulation of cellular gene expression which may promote cell growth and pathogenesis.

## Author Summary

Understanding how a persistent virus like Hepatitis C Virus (HCV) interfaces with the cellular machinery during infection can provide significant insights into mechanisms of pathogenesis. We demonstrate that while trying to degrade HCV transcripts, a major cellular exonuclease called XRN1 stalls and gets repressed in the 5’ noncoding region of the viral mRNA. Interestingly, the region where XRN1 stalls in the 5’ UTR includes the viral IRES that is required for translation initiation, uncovering a novel, unexpected function for this well-studied region. Differential mRNA stability is a major regulator of gene expression in cells. Curiously, repression of the cellular XRN1 exonuclease is associated with a general repression of mRNA decay in general in HCV-infected cells. Thus numerous cellular mRNAs are stabilized and accumulate in a dysregulated fashion during HCV infection. Normally short-lived mRNAs are disproportionately affected—including mRNAs that encode immune regulators and oncogenes. Thus, this study suggests a novel role for the 5’ UTR of HCV in molecular pathogenesis—including hepatocellular carcinoma. Furthermore, the 5’ UTR of Bovine Viral Diarrhea virus also represses the XRN1 enzyme and stabilizes cellular mRNA. Therefore a strategy of 5’ UTR-mediated XRN1 repression appears to be conserved among the vector-independent members of the *Flaviviridae*.

## Introduction

Hepatitis C virus (HCV) is a positive-sense RNA virus of the *Hepacivirus* genus within the *Flaviviridae* that chronically infects approximately 130–150 million people worldwide. It has a 9.6 kb genome that encodes a single large polyprotein that is processed to form 10 proteins [1]. Chronic HCV infection causes acute liver dysfunction, cirrhosis and is associated with the development of hepatocellular carcinoma (HCC). From a molecular perspective, although several viral proteins have been suggested to contribute to HCV-associated carcinogenesis [2], it is not clear how infection with a simple RNA virus that does not encode any known oncogenes causes such a complex and heterogeneous cancer. Thus a deeper understanding of the molecular interaction between HCV RNA and protein components with cellular factors is needed to shed light on molecular mechanisms of viral pathogenesis.

In addition to encoding a large polyprotein from its single open reading frame, HCV contains complex untranslated regions (UTRs) at both the 5’ and 3’ ends. The 3’ UTR is capable of interacting with numerous cellular proteins and contains several regions that are important for viral translation efficiency [3–5], RNA synthesis [6] and providing communication between the 5’ and 3’ portions of the viral genome [7]. The 5’ UTR is better characterized and harbors four conserved stem loops (denoted as stem loops I-IV from the 5’-3’ direction) with several noteworthy features. First, while stem loops I and II are involved in replication [8], stem loops II-IV of the 5’ UTR (plus a short region of coding sequence) form a highly structured internal ribosome entry site (IRES) that is required for HCV translation initiation [9]. Second, the cellular small microRNA miR-122 interacts with two regions near the 5’ end of the HCV genomic RNA [10–15]. Instead of repressing gene expression as one might expect from a miRNA, miR-122 actually stimulates viral gene expression and replication. This appears to be due in part to miR-122 interactions with the 5’ UTR that prevent access of the viral RNA to the cellular 5’-3’ exoribonuclease XRN1 [16–18]. Since targeting miR-122 is currently in clinical trials as a potential anti-HCV therapy [19], it appears that the viral RNA on its own may be highly targeted by XRN1 during infection.

The regulation of cellular mRNA abundance is not solely dependent on transcriptional efficiency, but rather also relies on differential mRNA stability to determine the abundances of transcripts in response to cellular and environmental signals [20]. Studies have demonstrated that between ∼20 and 50% of gene expression may be regulated post-transcriptionally at the level of mRNA decay [21–26]. Furthermore, RNA decay also plays a major role in the quality control of cellular gene expression [27]. The major pathway of general mRNA decay is usually initiated by a deadenylation event followed by exonucleolytic decay of the body of the mRNA [28]. In the 5’-3’ decay pathway, deadenylated mRNAs are first decapped and the resulting 5’ mono-phosphorylated mRNA is rapidly degraded by the highly processive 5’-3’ exonuclease XRN1 [29,30]. In the alternative 3’-5’ pathway, deadenylated transcripts are attacked by either the exosome complex or the 3’-5’ exonuclease DIS3L2 [31]. The exonucleolytic decay of deadenylated mRNAs is highly efficient in mammalian cells and it is very rare to observe decay intermediates.

Given the importance of the cellular mRNA decay machinery in regulating the quality and quantity of gene expression, any perturbation of this machinery could have significant consequences for cellular physiology. Thus viral interference with the decay machinery—perhaps as a natural strategy to stabilize viral transcripts during infection—could cause major disruptions in cellular gene expression patterns. Studies are emerging to suggest that this could be a major strategy employed by a variety of viruses to both maintain efficient viral replication as well as perhaps contribute to viral-induced cytopathology by disrupting cellular gene expression [32].

Arthropod-borne members of the *Flaviviridae* (e.g. West Nile virus (WNV) and the Dengue viruses (DENV)) have been shown to contain a conserved structure at the beginning of their 3’ UTRs that plays an important role in viral biology [33]. This region of the 3’ UTR folds into an interesting three helix junction that stalls the cellular XRN1 enzyme as it tries to degrade flaviviral transcripts [34,35]. The stalling of XRN1 at this structure results in the accumulation of large amounts of a short 3’ UTR-derived subgenomic flavivirus ‘sfRNA’ during infection [36,37]. The generation of sfRNA appears to be important for the success of a flavivirus infection as it modulates host RNAi and interferon-responses [38–40]. Furthermore, generation of sfRNA represses the activity of XRN1 both in infected cells and in cell-free assays [41]. The generation of sfRNA and repression of XRN1 in WNV and Dengue virus type 2 (DENV-2) infections is associated with a significant stabilization of cellular mRNAs [41]. Interestingly, HCV and other non-arthropod associated members of the *Flaviviridae* such as the economically important Bovine Viral Diarrhea virus (BVDV) [42] do not generate an sfRNA-like molecule from their 3’ UTRs [36]. However, significant changes in cellular gene expression occur in both HCV and BVDV infections [43]. Thus it is unclear whether or how these viruses interface with XRN1 and the cellular mRNA decay machinery.

To address this question, in this study we demonstrate that HCV and BVDV contain structured regions in their 5’ UTR near or including the IRES region that both stall and repress XRN1 activity. XRN1 repression by the 5’ UTRs of these viruses can be demonstrated both in biochemical assays as well as in living cells. Interestingly, HCV or BVDV repression of XRN1 is associated with a dramatic repression of the major 5’-3’ decay pathway and a large increase in the stability and abundance of numerous normally short-lived cellular mRNAs. From a pathogenic standpoint, it is interesting to note that the mRNAs of many cellular oncogenes and angiogenic factors are significantly stabilized and increased in abundance during HCV infection. Therefore we conclude that XRN1 repression is a highly conserved and important facet of infections by disparate members of the *Flaviviridae*.

## Results

### XRN1 stalls during exonucleolytic decay of the 5’ UTRs of HCV and BVDV

**Fig 1:**
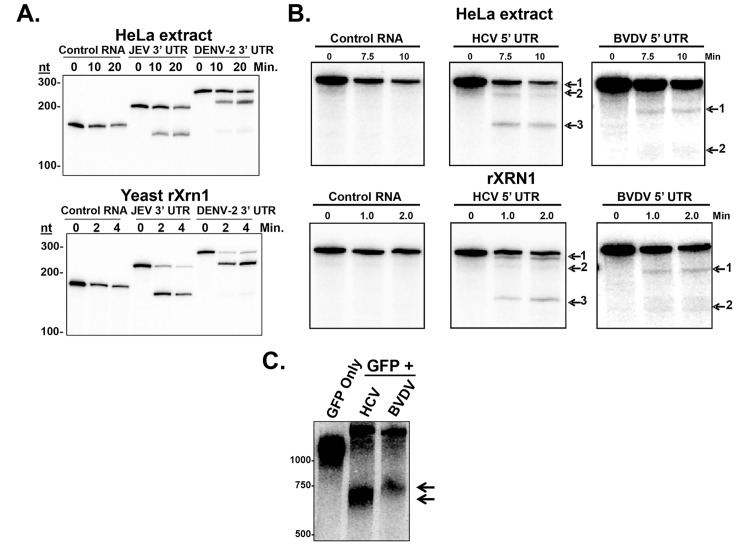
Stalling of XRN1 is a conserved function of flavivirus RNAs. Panel A. Radioactive RNAs derived from plasmid vector (control), or the 5’ portion of either the JEV 3’ UTR or DENV-2 3’ UTR were incubated in HeLa extract (top) or with recombinant XRN1 (bottom) under conditions that promote 5’-3’ decay. Products were analyzed on a 5% denaturing acrylamide gel. Panel B. Similar to Panel A, except the radioactive RNAs were derived from the 5’ UTR of HCV or the 5’ UTR of BVDV. Panel C. Human 293T cells were transfected with reporter plasmids expressing a standard GFP mRNA (GFP only) or a GFP mRNA containing either the 5’ UTR of HCV (HCV lane) or BVDV (BVDV lane) inserted into the 3’ UTR of the construct. Expressed RNAs were analyzed by northern blot using a probe to the 3’ region of the GFP mRNA (3’ of the viral RNA sequences). Arrows indicate decay intermediates.

Previous studies on arthropod-borne members of the *Flaviviridae* indicated that XRN1 stalls on specific structures at the proximal side of the 3’ UTR to generate a short RNA referred to as sfRNA [34,35]. As seen in Fig. 1A, XRN1 stalling/sfRNA generation can be recapitulated from the 3’ UTRs of Japanese Encephalitis virus (JEV) or DENV-2 in cell free systems using either HeLa cytoplasmic extracts (Fig. 1A upper panel) or purified recombinant XRN1 enzyme (Fig. 1A lower panel). While the generation of sfRNA by XRN1 in DENV-2 has been demonstrated several times [34,36,37,41], the biogenesis of sfRNA from JEV by XRN1 has not been formally documented. Importantly, recent collaborative work indicated that XRN1 stalling by these viral 3’ UTRs is due to novel interwoven pseudoknots around a conserved three-way junction [35].

Non-arthropod-borne members of the *Flaviviridae*, namely the hepaciviruses (e.g. HCV) and the pestiviruses (e.g. BVDV) do not make a detectable short sfRNA during infection from their 3’ UTRs [36]. Since XRN1 stalling is a conserved property of a large number of other members of the *Flaviviridae* [33], we hypothesized that HCV and BVDV might use structures elsewhere in their genomes such as the coding region or the 5’ UTR in order to stall XRN1. These HCV and BVDV 5’ UTRs, after all, are very highly structured and contain IRES elements that direct initiation of translation [44]. If this hypothesis were correct, XRN1-mediated decay intermediates that result from enzyme stalling on HCV and BVDV mRNAs would be nearly full length in size (∼10Kb), and thus difficult to detect without specifically assessing the 5’ end of these viral transcripts. Interestingly, a previously reported 5’ end analysis of HCV RNAs during infection by circularization RT-PCR revealed large numbers of HCV RNAs with 5’ ends that were truncated to around 55/56 and 80 nucleotides from the *bona fide* 5’ end [14]. These nested sets of 5’ shortened HCV mRNAs could be consistent with XRN1 decay intermediates generated by the stalling of the enzyme at structures in the 5’ UTR.

**Fig 2:**
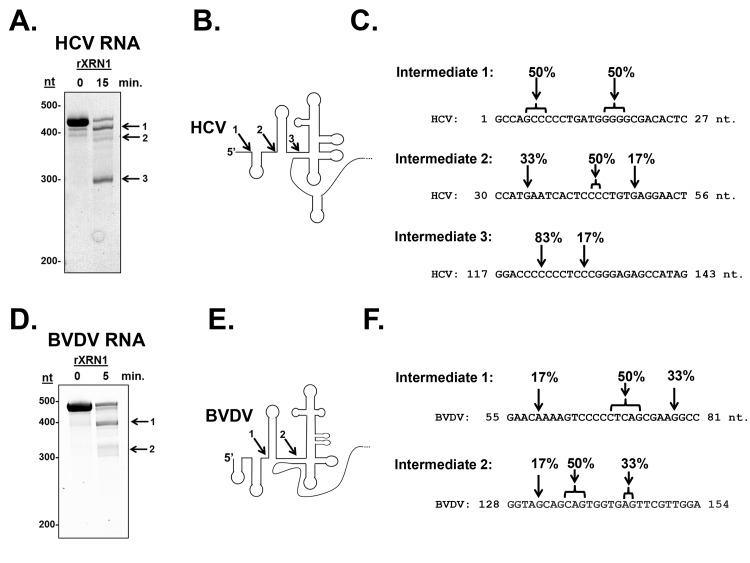
Determination of the 5’ ends of the HCV and BVDV 5’ UTR XRN1-mediated decay intermediates. Unlabeled RNAs containing either the HCV (Panel A) or BVDV 5’ UTR (Panel D) were incubated with recombinant XRN1. Decay intermediates were identified by SYBR green staining on a 5% denaturing acrylamide gel. The indicated bands were excised, circularized, and the 5’-3’ junction fragment was cloned and sequenced. Panels C and F: The position of the 5’-most nucleotide in each decay intermediates (1, 2, and 3) is indicated by the arrows. The percent of observed 5’ ends in the six sequenced clones from each RNA intermediate are indicated. Panel B and E. HCV and BVDV 5’UTR structure diagrams (modified from [46]) with arrows indicating the sites where XRN1 stalls. The numbers indicate which intermediate corresponds to the gels in panels A and D.

To formally test the hypothesis that XRN1 stalls in the 5’ UTR of HCV and BVDV, we created RNA substrates that have a 5’ mono-phosphate (and are therefore susceptible to XRN1 digestion) and contained either an HCV/BVDV 5’ UTR or control sequences. These RNAs were incubated in XRN1 exonuclease assays either using HeLa cytoplasmic extract (Fig. 1B upper panel) or purified recombinant XRN1 (Fig. 1B, lower panel) using incubation times to optimize 5’-3’ exonucleolytic decay. HeLa cytoplasmic extracts were used in these studies due to their well-characterized usage in RNA decay studies (e.g. [41]). However it should be noted that they may also contain XRN2 activity and this 5’-3’ exonuclease may also be contributing to the decay observed in these assays [45]. As seen in Fig. 1B, RNA substrates that contained either the HCV or BVDV 5’ UTRs generated XRN1-mediated decay intermediates. For HCV, three degradation intermediates were identified by cloning and sequencing with 5’ ends at approximately 5, 45 and 123 bases from the normal 5’ end of the genomic RNA. BVDV RNA degradation yielded two decay intermediates, a strong band at ∼70 nt and a weaker but reproducible stop at ∼137 nt from the 5’ end of the genome. The positions of these XRN1 decay stall sites on the HCV and BVDV 5’ UTRs are indicated in Fig. 2. Notably, the stall sites are all positioned at or near predicted sites of structural landmarks in both RNAs [46]. Importantly, no XRN1 decay intermediates are seen with numerous control RNAs in these assays (Figs. 1B, S1A and [47]). The observation of decay intermediates when using purified XRN1 and purified RNA implies that the RNA by itself is sufficient to stall XRN1 and no additional RNA-protein interactions are needed.

In order to confirm that the HCV or BVDV 5’ UTR by itself in the absence of other components of a viral infection in living cells could stall XRN1, we inserted the 5’ UTRs into a reporter downstream of a GFP open reading frame and transfected the expression constructs (or a control reporter that lacked any viral sequences) into 293T cells. A siRNA complementary to the GFP portion of the reporter RNA was added to cleave the transcripts and generate a large pool of RNA substrates available for XRN1 digestion in transfected cells [48]. As seen in the northern blots in Fig. 1C, while the control GFP construct did not generate any mRNA decay intermediates, both the HCV- and BVDV-containing GFP reporter constructs generated mRNA decay intermediates that contained viral RNA sequences. These intermediates were consistent with XRN1 stalling at or near domain II in the HCV 5’ UTR by RNase protection mapping of the 5’ end. The reason we see only one apparent decay intermediate likely includes gel resolution and/or changes in local RNA structure due to context/protein-RNA interactions on the reporter mRNA. Cell-type-specific alterations in the pattern of sfRNA decay intermediates from XRN1 stalling on the yellow fever virus 3’ UTR [37]. Therefore, we conclude that the 5’ UTRs of both HCV and BVDV transcripts contain sequences and/or structures that are capable of stalling the XRN1 enzyme. Thus, it appears that many if not all members of the *Flaviviridae* may use a related strategy of XRN1-refractory segments in an untranslated region of their transcripts to stall this highly processive cellular exoribonuclease.

### XRN1 activity is repressed by the 5’ UTRs of HCV and BVDV

**Fig 3:**
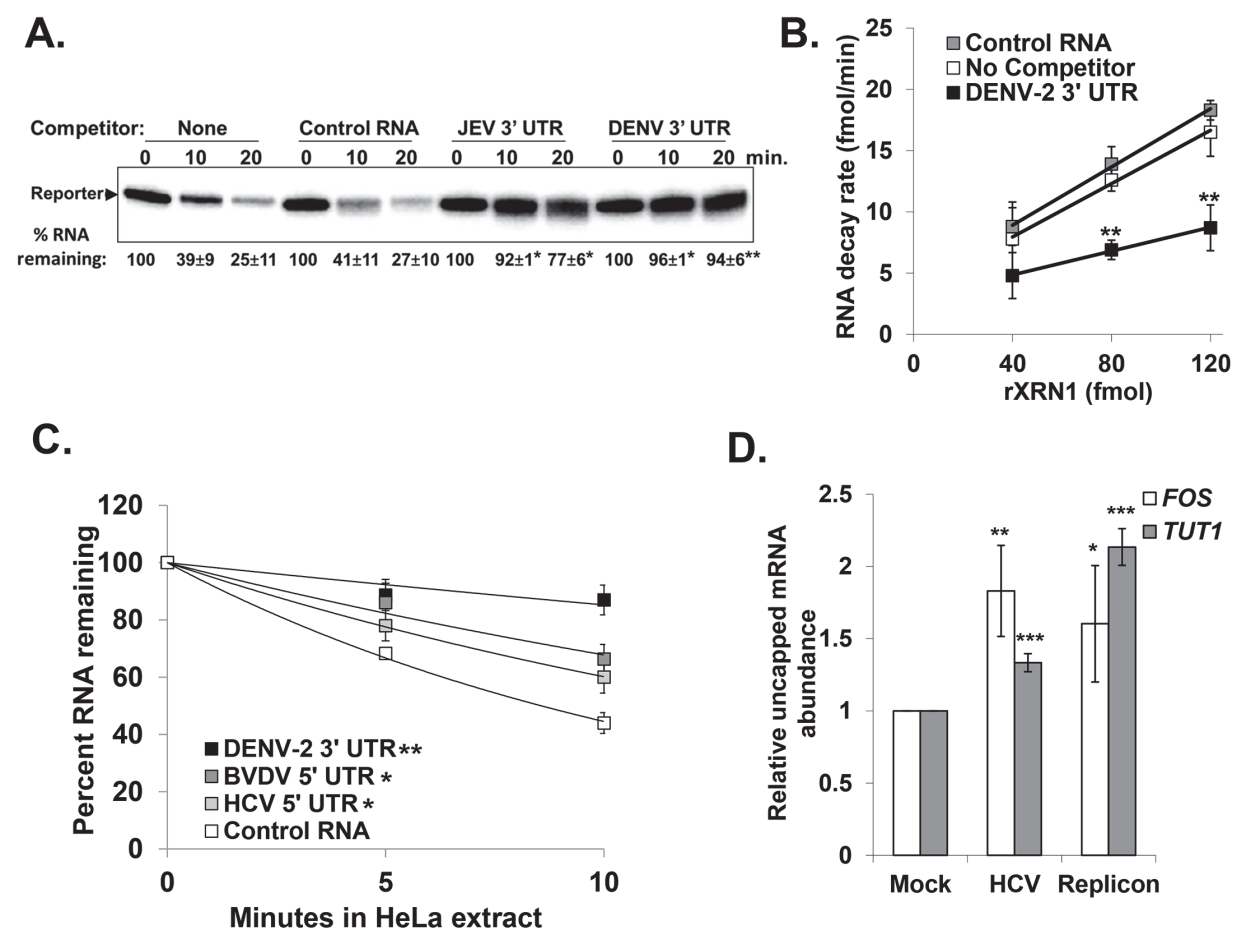
RNAs containing the 3’ or 5’ UTR of various members of the *Flaviviridae* are reversible inhibitors of XRN1 activity. Panel A. The effects of adding control (pGem-4 derived), JEV 3’ UTR, or DENV 3’ UTR competitor RNAs that contained a 5’ monophosphate on XRN-1 mediated decay of a radioactive reporter transcript in HeLa extract were assessed over the indicated time course of incubation. Products were analyzed on a 5% denaturing acrylamide gel and quantified by phosphorimaging. The average +/− standard deviation of the percent reporter RNA remaining from two independent experiments is shown below the gel. Asterisks indicate significant differences of percent reporter RNA remaining compared to controls. Panel B. Increasing amounts of recombinant XRN1 were incubated with a radioactive reporter transcript either in the absence of competitor RNA (No Competitor), with the DENV-2 3’ UTR competitor RNA (DENV-2 3’ UTR), or with a control competitor RNA (Control RNA). The rate at which the reporter RNA decayed in the presence of each competitor was calculated over the indicated range of XRN1 concentrations. The average +/− standard deviation of three independent experiments is shown with asterisks indicating significant differences between the reporter RNA decay rates compared to control at each time point. Panel C. Similar to panel A with the addition of the HCV and BVDV 5’ UTR RNAs included as competitors. Results were quantified as described for panel A and the average +/− standard deviation of three independent competition experiments is shown graphically. The significance of the differences in the half-life of the reporter RNA in the presence of DENV-2, BVDV, or HCV competitor RNAs compared to reactions containing the Control RNA competitor are indicated in the inset. Panel D. Total RNA from HCV replicon containing or HCV infected Huh7.5 cells was fractionated using an antibody to the methyl guanosine cap structure and qRT-PCR performed to detect *TUT1* and *FOS* mRNA abundances in the uncapped fraction using the endogenous *7SL* transcript as a reference gene. The uncapped fraction was normalized to 10% of the input RNA. The average +/− standard deviation of RNAs from three independent infections is shown. Significance was determined by t-test. For all panels *p≤0.05, **p≤0.01 and ***p≤0.001.

In addition to stalling XRN1, we previously demonstrated that XRN1 enzymatic activity is repressed by the generation of sfRNA from WNV or DENV-2 3’ UTRs [41]. These observations are now extended to include sfRNA generation from the 3’ UTR of JEV in Fig. 3A. Briefly, a radiolabeled reporter RNA containing a 5’ mono-phosphate was efficiently degraded by XRN1 in HeLa cytoplasmic extracts in the absence of any competitor RNA (‘none’ lanes) or in the presence of a control RNA competitor (‘Control RNA’ lanes). However, RNA competitors containing either the 3’ UTR of JEV or the 3’ UTR of DENV-2 dramatically repressed XRN1 activity and this sfRNA-mediated repression was reversible (Fig. 3B). When the rate of RNA decay was measured relative to increasing concentrations of XRN1 enzyme, the rate of the reaction slowed in the presence of an sfRNA generating competitor RNA relative to control conditions, but the enzyme was not effectively titrated away by the RNA inhibitor as would be expected for an irreversible competitor.

To assess whether sequences/structures in the 5’ UTR of HCV and BVDV that stall XRN1 can also repress its activity, similar competition assays were performed as in Fig. 3A. As seen in Fig. 3C, both HCV and BVDV RNAs significantly repressed XRN1 activity in HeLa cytoplasmic extracts compared to control RNA. While the XRN1 repression that was observed with the HCV or BVDV 5’ UTR competitors was substantial, it was consistently approximately half as robust as that observed with DENV-2.

To confirm that XRN1 can be repressed by HCV during infection, the relative levels of uncapped cellular mRNA were measured in naïve Huh7.5 cells, cells infected with HCV (JFH-1 strain) for 72 hours, or Huh7.5 cells that stably harbored an HCV replicon (with the H77 strain 5’ UTR) [49]. Uncapped mRNAs serve as a sensitive readout for XRN1 activity since they naturally represent a very small percentage (∼3%) of the total mRNA population in a cell. As seen in Fig. 3D, there was a significant increase in uncapped *FOS* and *TUT1* mRNAs (two representative short-lived transcripts) as a consequence of HCV infection. Furthermore, Huh7.5 cells harboring the HCV replicon RNA also had significantly more uncapped *FOS* and *TUT1* mRNAs than naïve control cells. Finally, the levels of Xrn1 protein remain unchanged in HCV infected cells (S1B Fig and [50,51]). Thus we conclude that XRN1 activity is repressed when it stalls while attempting to degrade the 5’ UTRs of HCV and BVDV.

### Cellular mRNA stability is altered when XRN1 activity is repressed by the HCV or BVDV 5’ UTR

**Fig 4:**
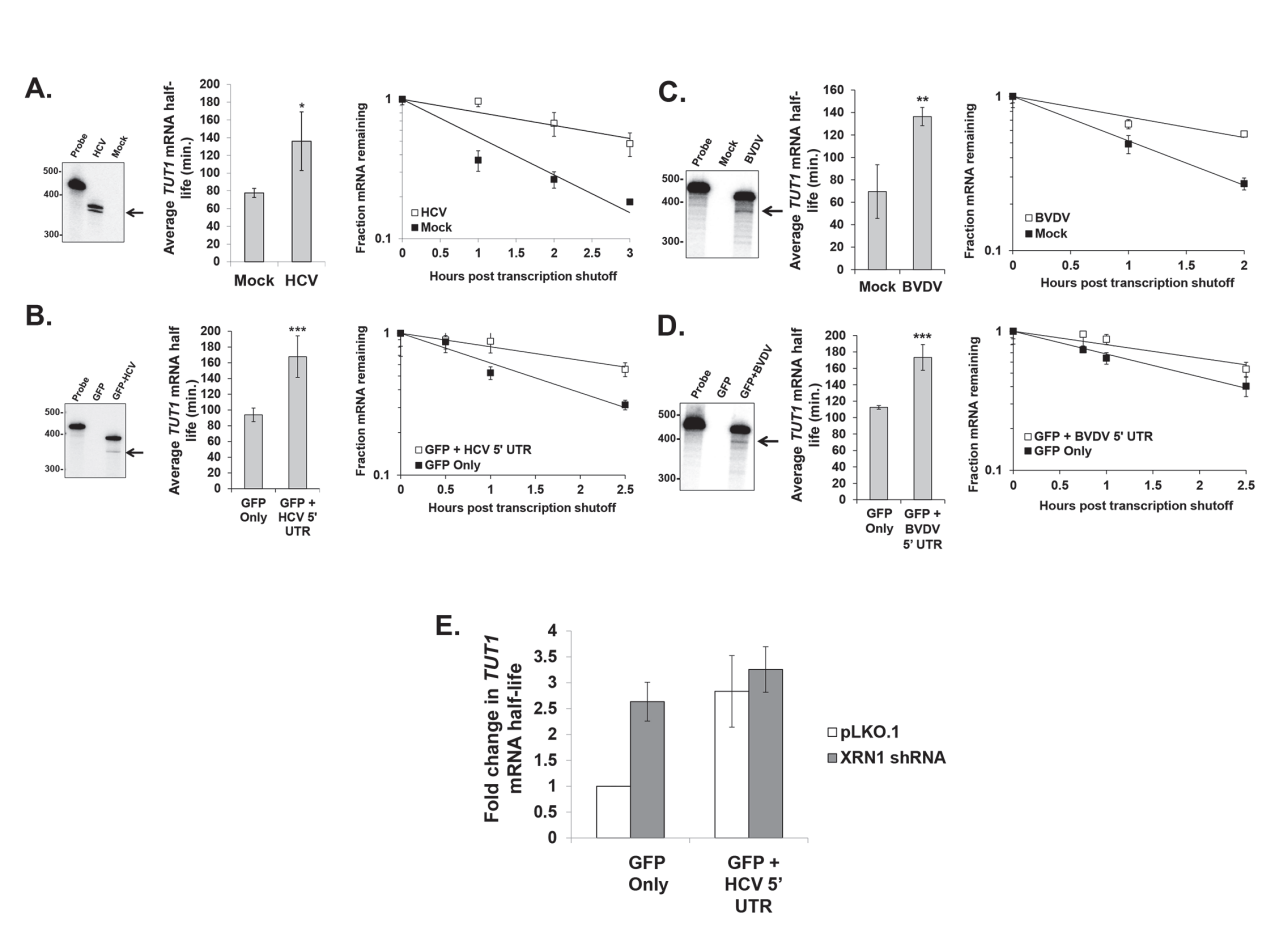
Cellular mRNA stability is similarly altered during HCV or BVDV infection or expression of reporter mRNAs containing the viral 5’ UTRs. Panel A. Assessment of *TUT1* mRNA stability using actinomycin D in human Huh7.5 cells infected with HCV compared to mock infected cells. Right panel: RNase protection analysis for HCV-specific RNAs. The arrow indicates the position of the likely decay intermediate generated by XRN1 stalling. Middle panel: the average TUT1 mRNA half-life +/− standard deviation determined from three infections. Right panel: a representative *TUT1* mRNA decay curve from HCV-infected cells and mock infected cells. Panel B. *TUT1* mRNA stability analysis as described in Panel A, but in transfected cells either expressing only a reporter mRNA (GFP Only) or a reporter RNA containing the 5’ UTR from HCV (GFP + HCV 5’ UTR) in 293T cells. Panel C. As described in panel A, but using BVDV infected MDBK cells. Panel D. As described in panel B, but using transfected 293T cells either expressing only a reporter mRNA (GFP Only) or a reporter RNA containing the 5’ UTR from BVDV (GFP + BVDV 5’ UTR). Significance in panels A-D was determined by t-test with *p≤0.05, **p≤0.01 and ***p≤0.005. Panel E: As described in Panel B, but cells were also transfected with either an empty vector control (pLKO.1) or with a vector expressing an XRN1-specific shRNA. The average fold change in *TUT1* mRNA half life +/− standard deviation from two independent co-transfection experiments is shown.

The repression of XRN1 activity by HCV or BVDV infection suggests that cellular mRNA decay may be dramatically dysregulated during viral infection. To test this hypothesis, we measured mRNA half-lives of a representative cellular mRNA (*TUT1*) in mock infected and HCV or BVDV infected cells. As seen in Fig. 4A & C (right panels), the *TUT1* mRNA was significantly stabilized during HCV or BVDV infection. Importantly, *TUT1* stabilization was observed in the presence of a truncated sfRNA-like HCV or BVDV RNA as determined by RNase protection assays using a probe that spanned the entire IRES element of each virus (Fig. 4A &C, left panels). To determine whether the stabilization of the *TUT1* mRNA could be directly attributed to XRN1 inhibition by the 5’ UTR of HCV or BVDV rather than other viral-specific aspects of infection, we transfected human 293T cells with reporter constructs expressing either GFP mRNA only or a GFP reporter mRNA containing viral 5’ UTR sequences inserted downstream of the open reading frame. We placed the viral 5’ UTR at this downstream location for two reasons. First, a *bona fide* RNA domain that stalls XRN1 should function regardless of where it is placed in a transcript. Second, placement near the 3’ end provided for easy detection of relatively short decay intermediates on standard acrylamide gels. 293T cells were used in this experiment due to their high transfection efficiency. As seen in Fig. 4B & D (right panels), *TUT1* mRNA was significantly stabilized (approximate two-fold increase in mRNA half-life) by the reporter RNA containing the HCV or BVDV 5’ UTR sequence in association with the build-up of truncated sfRNA-like reporter RNAs (left panels). Thus, we conclude that the presence of RNAs containing the HCV or BVDV 5’ UTR has a dramatic effect on *TUT1* mRNA stability in host cells.

Finally, we performed a similar transfection experiment using the GFP mRNA constructs +/− the HCV 5’ UTR in 293T cells that were also transfected with either an empty vector (pLKO.1) or a vector expressing an shRNA to knock down XRN1. As seen in Fig. 4E, knockdown of XRN1 on its own results in the significant stabilization of the cellular *TUT1* mRNA (p<0.05; see also [41]). Furthermore, when XRN1 knockdown cells are transfected with the GFP construct containing the HCV 5’ UTR, we failed to observe any significant further increase in *TUT1* mRNA stability as is observed when cells are treated with a control shRNA vector (pLKO.1). Collectively, these data suggest that the increase in cellular mRNA stability that is associated with the presence of the HCV 5’ UTR requires XRN1.

### Global analysis of host mRNA decay rates during HCV infection

**Fig 5:**
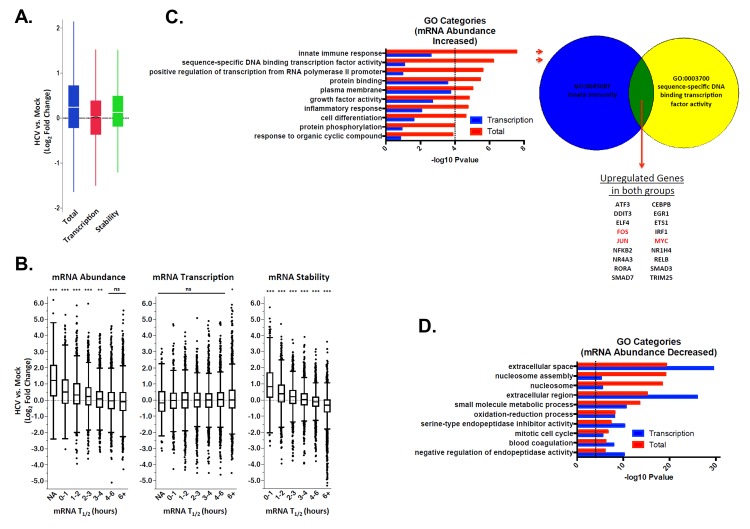
Significant increases in global cellular mRNA expression observed in HCV infection are largely due to increased mRNA stabilization. Mock-infected or HCV-infected Huh7.5 cells were pulse labeled with 4sU. Total RNA was fractionated into labeled and unlabeled fractions and subjected to RNAseq analysis. Transcription and mRNA decay rates were then determined and compared. Panel A: Raw fold changes (log2) in mRNA abundance (Total), transcription rates (Transcription) and mRNA half-lives (Stability) in HCV infected cells compared with naïve Huh7.5 cells. Panel B: Normalized fold changes (log2) in total gene expression (left panel) transcription (center panel) and RNA stability (right panel) in mock versus HCV-infected cells were graphed relative to mRNA half-life prior to infection. NA indicates a population of mRNAs with a normalized half-life calculated to be negative (suggesting highly unstable mRNAs). Significance was calculated using ANOVA and Tukey’s HSD test; * p <0.05, ** p <0.01, *** p<0.001. Panel C. GO analysis to identify categories of genes that were up-regulated in HCV infection relative to mock infected cells in the total dataset (red bars) or only in the transcription analysis (blue bars). The proportion of the abundance increase that is not due to transcription is likely due to RNA stabilization. To the right of the graph is an illustration depicting genes that are up-regulated in both of the top two GO categories. *FOS*, *JUN* and *MYC*, which are followed up in the study, are highlighted in red. Panel D. GO analysis for genes that are down-regulated in HCV infection relative to mock infected cells in the total dataset (red bars) or only in the transcription analysis (blue bars).

In order to expand the observation of viral 5’ UTR-induced repression of XRN1/mRNA stabilization, we measured global mRNA decay rates in mock infected and HCV infected Huh7.5 cells after 120 hours at which point the infection rate was ∼70%. One hour prior to RNA extraction, cells were pulse labeled with 4-thiouridine (4sU) to mark nascent transcripts. Three populations were isolated and sequenced: total RNA, 4sU-labeled nascent transcripts and unlabeled transcripts. Categorizing the mapped reads from each population revealed three important features: nascent transcripts were enriched for pre-mRNA, as seen by the increase in the presence of intronic reads (S2A Fig); HCV infection did not appear to interfere with global mRNA processing in the infected cell (S2A Fig); and the relative abundances of RNA in each replicate sample clustered well (S2B Fig). These analyses suggest that we generated a high quality data set for further examination. Total mRNA abundances, transcription rates and half-lives were determined as previous described [52,53] and compared between mock and HCV infected samples (S4 Table). Importantly, the changes in specific mRNA levels that we observed correlated well with a data set obtained at similar times of HCV infection by Walters et al. [43] (S2C Fig). Interestingly, in both replicates we observed an overall increase in cellular mRNA abundance in HCV infected cells compared to mock infected cells without a concomitant increase in transcription rate prior to normalization (Fig. 5A). Rather, there was a global increase in mRNA stability directly associated with the global increases in mRNA abundance seen in HCV infected cells (Fig. 5A). A broad increase in mRNA abundance caused by XRN1 repression would be expected to affect mRNAs with short half-lives more dramatically than mRNAs with long half-lives. As shown in Fig. 5B, the increase in mRNA abundance scaled directly with the stability of the mRNA prior to infection. For all mRNAs with calculated half-lives of less than 4 hours, each progressive decrease in stability resulted in a significant increase in abundance. This was associated with similar progressive increases in stability but not transcription (Fig. 5B, center and right panels). Thus, we conclude that HCV infection causes a significant dysregulation of mRNA stability whose effects are most strongly observed in mRNAs that have a short half-life in uninfected cells.

We further investigated the Gene Ontology (GO) of differentially regulated mRNAs in mock versus infected cells using InnateDB [54]. RNAs that increased in abundance were enriched for innate immune responses and transcription factors (Fig. 5C). These functional groups tend to consist of unstable mRNAs [55,56]. Interestingly, none of the GO categories that were significantly enriched in the up-regulated mRNA abundance dataset reached significance in the up-regulated mRNA transcription dataset (Fig. 5C). By comparison, all GO categories significantly enriched in down-regulated mRNA abundance were also significant in the down-regulated mRNA transcription set (Fig. 5D). This suggests decreases in mRNA abundance are primarily transcriptionally driven, while increases in mRNA abundance are not.

**Fig 6:**
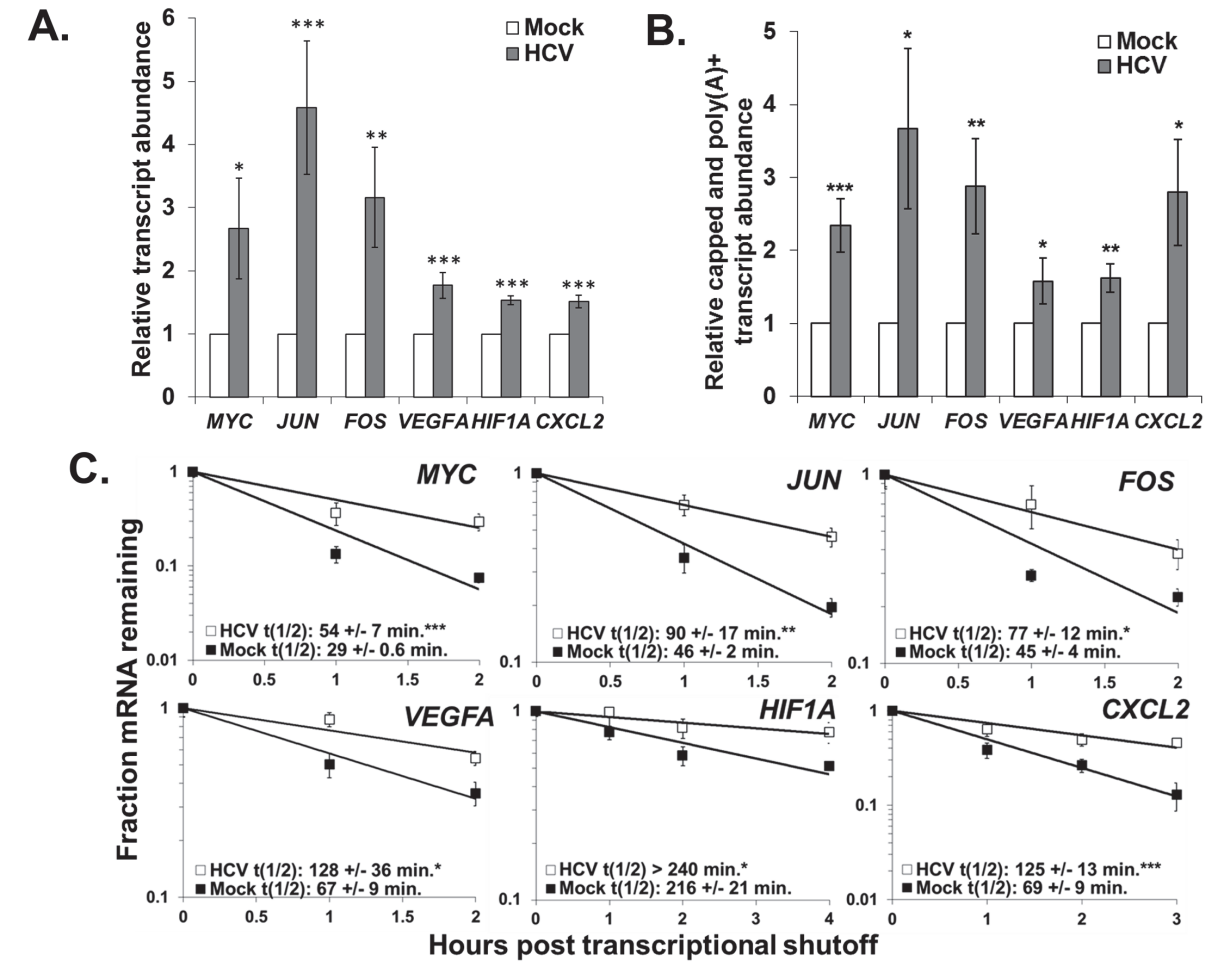
Hepatitis C virus infection induces up-regulation of potent oncogenes and angiogenic factors in Huh7.5 cells. Panel A. Huh7.5 cells were mock-infected or infected with HCV. Total RNA was isolated and the abundance of the indicated mRNAs was determined by qRT-PCR using *GAPDH* mRNA levels for normalization. The average +/− standard deviation of each transcript level from three infections is reported. Panel B. Total RNA from mock- or HCV-infected cells was fractionated using an antibody to the methyl guanosine cap structure and the relative abundance of each mRNA in the capped mRNA fraction was determined by RT-qPCR using oligo dT as a primer for the RT reaction to selectively amplify polyadenylated mRNAs. *GAPDH* mRNA was used as a reference for normalization and the average relative transcript abundance +/− standard deviation from three infections is shown. Panel C. Representative mRNA decay curves for the indicated transcripts as determined by qRT-PCR analysis of total RNA samples obtained at the indicated times post actinomycin D shut off of transcription. The average half-life +/− standard deviation from three infections is reported in the graph insets. Significance in each panel was determined by t-test with. * p ≤ 0.05, **p≤0.01, ***p≤0.005.

To confirm our observations regarding dramatic increases in mRNA abundance and stabilization in HCV infected cells, we repeated mRNA half-life analysis with a more classical actinomycin D transcriptional shut off approach in mock-treated or HCV infected cells and analyzed changes in RNA abundance over a time course using qRT-PCR assays. We selected unstable innate immunity and transcription factors, focusing on three oncogenes (*MYC*, *JUN* and *FOS*) and three angiogenic factors (*VEGFA*, *HIF1A* and *CXCL2*). Since HCV infection is associated with the development of hepatocellular carcinoma, these factors might contribute to the development of a diseased state. As seen in Fig. 6 and in S1 and S2 Tables in S1 Text, all six of these mRNAs were significantly increased in abundance 2–4 fold (panel A) as well as significantly stabilized (mRNA half-lives increased ∼2-fold) (panel C) in HCV infection after 72 hours. Finally, Gene Set Enrichment Analysis (GSEA) demonstrated a significant enrichment in upregulated mRNAs in HCV infection for genes that have promoter binding sites for AP-1 (JUN and FOS heterodimer) and MYC (S4 Fig). Thus the stabilization of these oncogenic transcription factor mRNAs also appears to lead to the generation of functional protein which further dysregulates cellular gene expression during viral infection.

To further assess whether the increases in abundance and stability we observed reflected functional mRNAs rather than the accumulation of non-functional uncapped mRNA decay intermediates resulting from XRN1 repression, we determined the relative levels of capped and polyadenylated mRNAs in mock versus HCV infected cells. As shown in Fig. 6B, there was a significant 2–3 fold increase in the abundance of intact and presumably fully translatable levels of mRNAs of the six oncogene and angiogenic factors assayed above. Thus, in addition to significant increases observed in the minor population of uncapped mRNAs in infected cells (Fig. 3D), there was also a dramatic increase in intact mRNAs (the major mRNA species) due to infection. We therefore suggest that by repressing XRN1 during HCV infection, a feedback mechanism takes place that appears to repress the entire 5’-3’ decay pathway, preventing the initial steps of decay that involve deadenylation and decapping.

### Cellular mRNA stability is also altered when XRN1 activity is repressed by the BVDV 5’ UTR

**Fig 7:**
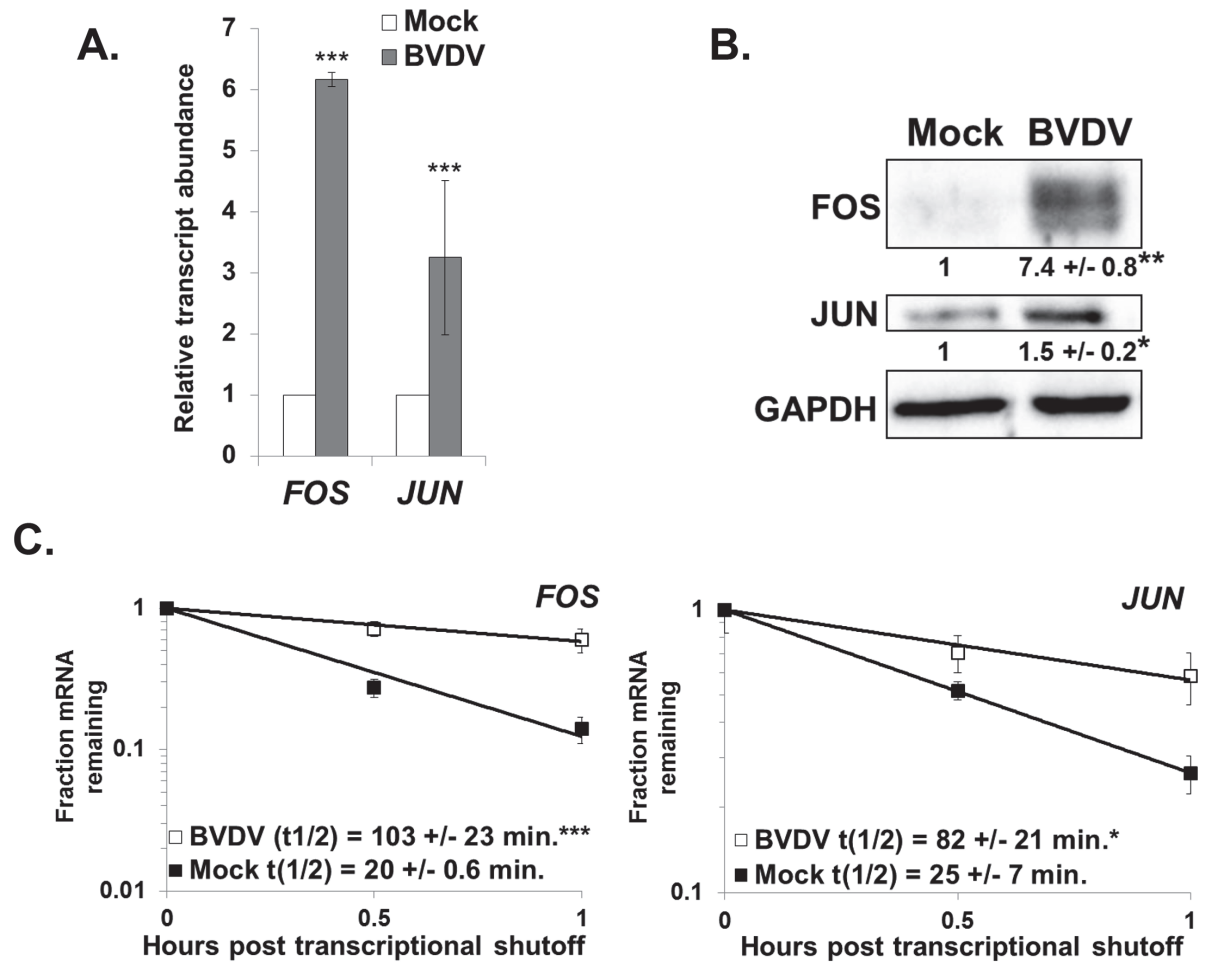
Changes in gene expression also indicate XRN1 suppression and increased mRNA stability during BVDV infection. Panel A. MDBK cells were either mock infected or infected with BVDV. Total RNA samples were assayed for the indicated cellular mRNAs by qRT-PCR using *ACTB* as a reference transcript. The average relative abundance of *FOS* and *JUN* mRNAs from three independent infections +/− standard deviation is depicted. Panel B. Western blot analysis of total protein samples using antibodies against FOS, JUN, or GAPDH. The average quantification of the blot (relative to GAPDH) +/− standard deviation from three infections is shown. Significance was determined by t-test with * p≤0.01; **p≤0.001. Panel C. mRNA half-lives were determined using actinomycin D for the *FOS* and *JUN* mRNAs in mock-infected or BVDV-infected MDBK cells. A representative mRNA decay curve is depicted with the average +/− standard deviation of each half-life from three independent infections shown in the graphical insets. Statistical analysis was performed using a t-test with * p ≤ 0.05 and ***p≤0.005.

Finally, to generalize our observations regarding dysregulation of cellular mRNA stability in an HCV infection to another non-arthropod-borne member of the *Flaviviridae*, we assessed the abundance and stability of select cellular mRNAs in BVDV infected MDBK cells. As shown in Fig. 7A, the abundance of both *FOS* and *JUN* mRNAs was significantly upregulated (three to six fold) in a BVDV infection. Furthermore, the proteins encoded by these mRNAs were also upregulated in a BVDV infection (Fig. 7B) (and a similar increase in protein expression was seen in HCV infection (S4 Fig). As seen in Fig. 7C, the increase in abundance of the *FOS* and *JUN* mRNAs can largely be accounted for by the approximately five and three-fold significant increase in mRNA half-life in a BVDV infection, respectively. Importantly, XRN1 levels are not significantly changed during BVDV infection (S1B Fig). Thus, we conclude that dysregulation of cellular mRNA stability is a common occurrence in hepacivirus and pestivirus infections and may play a heretofore unforeseen role in the success of viral replication as well as viral-induced pathogenesis.

## Discussion

**Fig 8:**
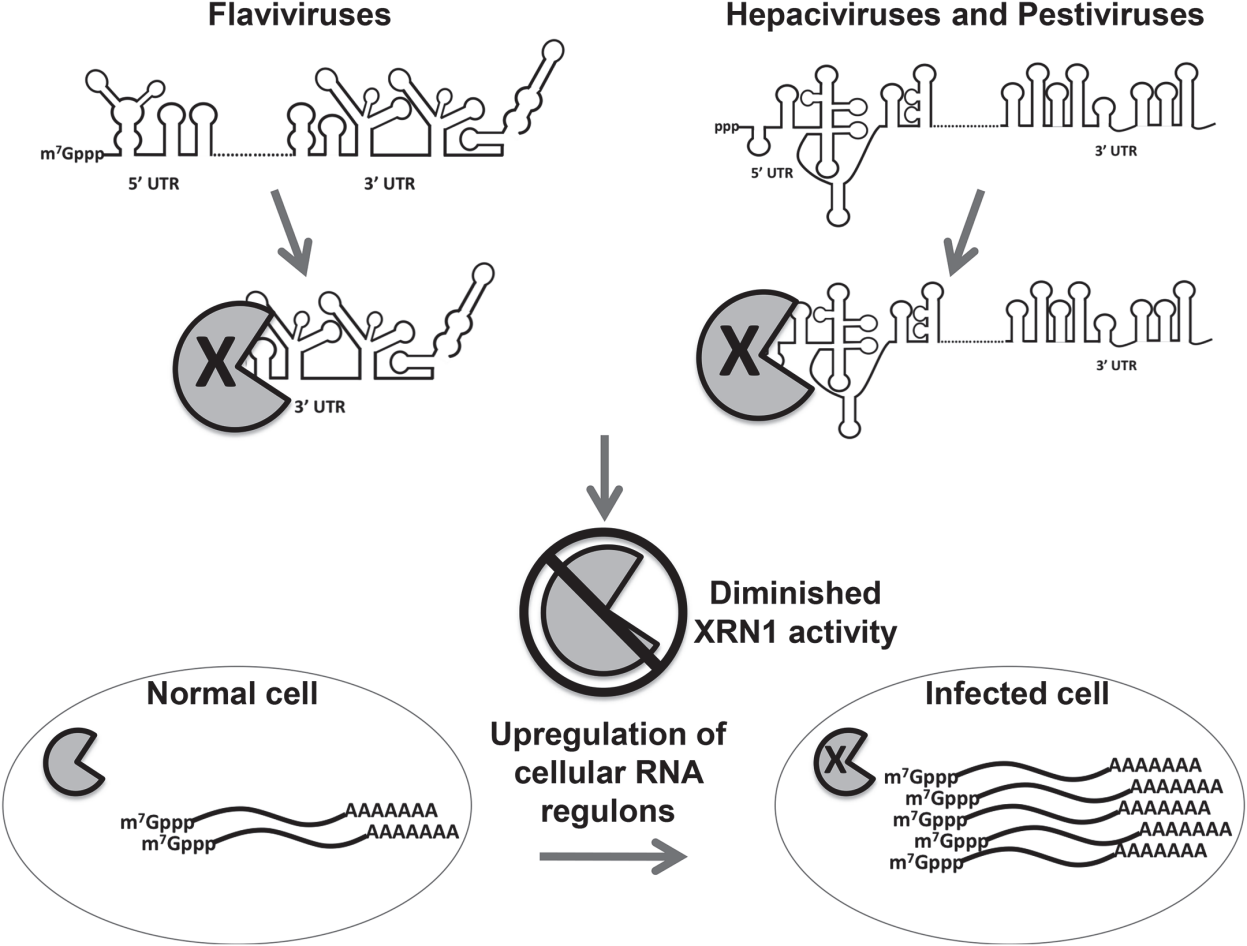
Model for the interaction and consequences of repression of XRN1 mediated by either the 5’ or 3’ UTRs of members of the *Flavivirdae*. The pacman structure indicates XRN1. When the exonuclease XRN1 begins degrading flavivirus RNAs in a 5’-3’ direction, it is stalled and repressed by structures in the either the 5’ or 3’ untranslated regions depending on the infecting virus. Repression of XRN1 activity results in an apparent shut down of the major 5’-3’ mRNA decay pathway in the infected cell, resulting in the significant stabilization and accumulation of many short lived cellular mRNAs. This dysregulation of cellular gene expression has a variety of potential implications to mechanisms of viral-induced cytopathology and pathogenesis.

In addition to the well-described role of the 5’ UTR IRES element in translation [9] and the binding of miR-122 to two sites in the HCV 5’ UTR to influence RNA stability [13,57], the data described in this study uncover an additional, unexpected role for the 5’ UTRs of HCV and BVDV in repressing the cellular XRN1 exoribonuclease. These observations indicate that the stalling/repression of XRN1 by a non-coding region in the genomes of members of the *Flaviviridae* is an evolutionarily conserved strategy for promoting the successful outcome of an infection. The stalling of XRN1, its subsequent repression and the implications of repressing this major cellular mRNA decay enzyme on gene expression in infected cells is summarized in Fig. 8. This conserved aspect of flavivirus infections suggests a potentially attractive antiviral target for therapeutics that may have broad-spectrum activity against members of the *Flaviviridae*.

Arthropod-borne members of this virus family use structures in their 3’ UTRs to perhaps generate large amounts of a non-coding sfRNA to provide additional functions such as modulating the invertebrate RNAi anti-viral response [33]. Large amounts of sfRNA produced in these infections could also allow an optimal interaction between the virus and its insect vector, including cellular protein binding/sequestration [58]. The selective pressure from the RNAi pathway, however, is likely not as great for the *Flaviviridae* members that do not use insect infections as part of their transmission cycle [40]. Thus, viruses like HCV and BVDV can readily use structures in the 5’ UTR instead of structures in their 3’ UTR, resulting in larger, nearly intact XRN1 decay intermediates when compared to the plus-sense genomic RNA. This strategy, particularly with the presence of an IRES element that has its main structural features localized to the distal half of the 5’ UTR [9], may maintain additional functionality to the XRN1-decay intermediates that are generated, perhaps including translatability.

While the unique three-helix junction structure that stalls XRN1 on the 3’ UTR of flaviviruses has recently been solved [35], the relationship with the structure in the 5’ UTR of HCV and BVDV that blocks the enzyme await further study. The RNA structure around the IRES region is rather complex in both HCV and BVDV, including pseudoknots that are also a hallmark of flaviviral 3’UTR structures [59]. In addition, an extended set of small conserved nucleotide blocks that was noted among XRN1-stalling region of the insect-borne flaviviruses [35] can also be identified in the HCV 5’ UTR. Another interesting question for future analysis is whether stalling of XRN1 is a general property of all or at least some other viral IRES elements. If so, this may reveal an interesting general strategy used by RNA viruses to effectively address two major, often functionally interrelated aspects of post-transcriptional gene expression in the cytoplasm—translation initiation and RNA stability. XRN1 repression, however, is not a strategy used by all RNA viruses. In our previously reported studies with Sindbis virus [60], we see no evidence of massive stabilization of cellular mRNAs as we do with HCV infections. In fact, we see mRNA destabilization due to the usurping of the cellular HuR factor by Sindbis virus.

The presence of miR-122, which interacts with two regions near the proximal end of the HCV 5’ UTR [13], could possibly have an influence on XRN1 repression and will require additional experimentation. However based on the ability of the HCV 5’ UTR to repress the activity of XRN1 in HeLa cytoplasmic extracts which do not contain appreciable amounts of miR-122 (Fig. 3), at this juncture we favor a model based on the available literature that the interaction of miR-122 with the HCV 5’ UTR makes the viral RNA refractory to XRN1 loading (either directly or by inhibition of an unidentified cellular phosphatase that acts on the 5’ trisphosphate), resulting in viral RNA stabilization. In this model, any population of HCV RNA that does not effectively interact with miR-122, however, would be a substrate for initiation of XRN1-mediated decay and would contribute to the stalling and repression of the cellular enzyme. Thus the HCV 5’ UTR may have a bipartite strategy for interfacing with XRN1—ensuring the availability of intact genomic RNA for replication and packaging through miR-122 interaction while allowing a portion of its RNA to lure the enzyme to initiate decay in order to repress XRN1 activity. Ultimately, this repression of XRN1 dysregulates cellular mRNA decay and likely prevents or significantly alters changes in gene expression which promote the proper cellular response to infection.

Repressing XRN1 appears to also repress the deadenylation and decapping aspects of the 5’-3’ mRNA decay pathway, resulting in the increased accumulation of capped and polyadenylated mRNAs during HCV infection. We envision the apparent shut down of the entire decay pathway by the 5’ UTR of HCV/BVDV could be due to at least three mechanisms (or a combination thereof). First, XRN1 has been shown to physically interact with the DCP1 component of the decapping complex [61]. Thus XRN1 repression by stalling on the 5’UTR of viral RNAs could also sequester/alter the localization of the decapping complex in an unproductive fashion. Likewise, deadenylation could be physically co-localized with XRN1 as well through interactions between hPAT1, the CAF1-CCR4-NOT deadenylation complex and the decapping complex [62]. Second, flavivirus infections, including HCV, have been shown to disrupt P bodies [50]. The disruption of these localized sites of aggregated mRNA decay components could contribute to the shutdown of the entire 5’-3’ decay pathway. Finally, XRN1 has been recently implicated as a key factor in mediating the buffering between the rates of transcription and mRNA decay for genes in order to maintain appropriate levels of gene expression in yeast [63,64]. Thus it is possible that by repressing XRN1, HCV and other flaviviruses are disrupting this natural aspect of buffering in cellular gene expression for at least some transcripts that ultimately contributes to their increased abundance.

Did flaviviruses independently evolve the ability to stall XRN1, or do perhaps select subsets of cellular mRNAs possess XRN1-refractory structures that allow the generation of RNAs with novel 5’ ends that may have independent functions from their parent mRNAs? Note that the XRN1 decay intermediate observed in Fig. 4 were generated from a *bona fide* mRNA made from a transfected reporter plasmid. Since most cellular RNAs would be vastly less abundant than viral RNAs in the cell, the amount of repression of XRN1 by the reversible, presumably slow release of the stalled enzyme on these RNAs observed in cells would likely be minimal and perhaps worth the expense in cells to generate novel RNAs.

The dysregulation of cellular mRNA stability and the associated increase in the abundance of numerous usually short lived mRNAs has significant implications for pathologies associated with HCV infection. Chronic HCV infection has been strongly linked to development of HCC. Along these lines, it is interesting to note that XRN1 has been previously implicated as a possible tumor suppressor gene in osteosarcoma [65]. While the causes of HCC are likely complex, involving inflammatory and fibrotic responses to the injured liver, dysregulated expression of short-lived mRNAs in HCV-infected cells leading to elevated expression of oncogenic factors may contribute to HCC.

## Materials and Methods

### XRN1 biochemical assays

*In vitro* XRN1-mediated RNA decay assays were performed using internally radiolabeled, 5’ monophosphorylated RNAs as previously described [41]. The 200nt fragment of the JEV 3’ UTR, the 218nt portion of the DENV-2 3’ UTR, the 389nt HCV 5’ UTR, the 440nt BVDV 5’ UTR and control RNAs used in study are described in the Supplementary methods (S1 Text).

### RNA analyses in cells, viral infection and transfection studies

Huh7.5, MDBK and HEK 293T cells were used in the study for HCV infection, BVDV infection and transfection analyses respectively. For transfections, the HCV or BVDV 5’ UTR were subcloned into the NotI site of peGFP-N1 and introduced into cells using Lipofectamine 2000. Total RNA was isolated with TRIzol and RNAs were quantified by qRT-PCR, northern blotting or RNase protection as described in detail in the Supplementary methods. mRNA abundance, synthetic rate and stability were determined using actinomycin D shut off and RT-qPCR or metabolic 4sU labeling and RNAseq as described in detail in the Supplementary methods.

### Analysis of capped/uncapped and polyadenylated mRNAs

Total RNA from naïve Huh7.5 cells, HCV infections or Huh7.5 cells harboring a replicon HCV construct was fractionated into capped and uncapped pools using an antibody that recognizes the methyl-guanosine cap structure (Synaptic Systems) as described in Moon et al. [41]. Please see the Supplementary Methods section of S1 Text for additional details.

### Western blotting

Western blotting was performed as described in Barnhart et al. [60] as outlined in the Supplementary methods.

## Supporting Information

S1 Text S1–S3 Tables and supplemental experimental procedures. **S1 Table.** Primers used for qRT-PCR. Related to Figs. 4, 5 and 7. **S2 Table.** Select average mRNA half-life calculations using either actinomycin D transcriptional shut-offs (72 hpi) or metabolic labeling with 4-thio uridine (4sU) (120 hpi). Related to Figs. 4 and 5. The average of three independent experiments is reported for actinomycin D shut-offs; average of two independent experiments is reported for metabolic labeling with 4sU. The fold change in mRNA half-life in mock vs HCV infected cells is shown in the ‘Fold change’ column. **S3 Table.** Fold increases in select mRNA abundances in HCV infected cells compared to mock infected cells as calculated by multiple methods. Related to Figs. 5 and 6. The average fold-change in mRNA abundances from three independent untreated RNA experiments using qRT-PCR analysis and two independent experiments using RNAseq.
(DOCX)

S1 Fig XRN1-mediated decay intermediates are not commonly observed during RNA degradation and XRN1 protein levels do not appreciably change in HCV or BVDV infections. Related to Figs. 1 and 4. Panels A. A series of radiolabeled RNAs containing a 5’ monophosphate was incubated with recombinant XRN1 for 0, 30 or 60 seconds. Reaction products were analyzed on a 5% denaturing acrylamide gel. Panel B. XRN1 levels in Huh7.5 cells (left panel) or MDBK cells (right panel) were analyzed by western blotting during mock or either HCV (left) or BVDV (right) infection. Average XRN1 levels from three HCV infections +/− standard deviation relative to RPL19 control are shown. Tubulin (TUBA1A) was used as a loading control in the gel on the right (representative of blots from three independent infections).
(TIF)

S2 Fig Validation of global RNAseq dataset. Related to Fig. 5. Panel A. HCV and Mock infected Huh7.5 cells were subjected to 4sU labeling for one hour prior to harvest, then RNA was separated into 4sU labeled, unlabeled and total populations and subject to Illumina sequencing. Tophat2 mapped reads were categorized as intronic, coding sequence, intergenic, UTR or HCV using PicardTools CollectRNASeqMetrics function. Reads mapping to the HCV genome in infected samples accounted for ∼1% of all mapped reads. Error bars represent standard deviation. Panel B. Normalized log2 transformed sequencing data correlated highly and samples clustered together. Panel C. Changes in mRNA abundance correlate well with previously published HCV infections. A total of 612 differentially expressed mRNAs from Walters et al. [43] at time points from 24–120 hours were compared to mRNA abundance changes observed in Mock versus HCV infected samples at 120 hours.
(TIF)

S3 Fig Comparison of the different HCV-derived 5’ UTRs used in the study. Related to Figs. 1–6. Clustal Omega alignment [S9, S10] of the sequences of the 5’ UTRs of the three strains of HCV used in the study. JFH-1 is the wild-type HCV infectious virus used in Figs. 2D, 3A, 4, and 5. The H77 5’ UTR was cloned into pGEM-4 and peGFP-N1 vectors for Figs. 1, 2, and 3; and the Replicon was used for Fig. 2D. Note that the various 5’ UTRs are over 92.5% similar to each other (determined by the clustal 2.1 percent identity matrix).
(TIF)

S4 Fig GSEA analysis of stabilized transcription factors. Related to Figs. 5–7. MSigDB datasets defining mRNAs with binding sites matching a set of predicted binding motifs for the FOS and JUN heterodimer AP-1 (left panels) and MYC (right panels) were evaluated for correlation with rank ordered changes in mRNA abundance (top panels) and mRNA transcription rate (bottom panels).
(TIF)

S4 Table Excel file with all half-lives and abundance data from global analysis of gene expression in HCV infections. Related to Figs. 5 and 6.
(XLSX)
