# Supplementary material for: XRN1 Stalling in the 5’ UTR of Hepatitis C Virus and Bovine Viral Diarrhea Virus Is Associated with Dysregulated Host mRNA Stability
Source: PLoS Pathog. 2015 Mar 6;11(3):e1004708. doi: 10.1371/journal.ppat.1004708 (PMC4352041; doi:10.1371/journal.ppat.1004708)
Supplement: S1 Text — S1 Table. Primers used for qRT-PCR. Related to Figs. 4, 5 and 7. S2 Table. Select average mRNA half-life calculations using either actinomycin D transcriptional shut-offs (72 hpi) or metabolic labeling with 4-thio uridine (4sU) (120 hpi). Related to Figs. 4 and 5. The average of three independent experiments is reported for actinomycin D shut-offs; average of two independent experiments is reported for metabolic labeling with 4sU. The fold change in mRNA half-life in mock vs HCV infected cells is shown in the ‘Fold change’ column. S3 Table. Fold increases in select mRNA abundances in HCV infected cells compared to mock infected cells as calculated by multiple methods. Related to Figs. 5 and 6. The average fold-change in mRNA abundances from three independent untreated RNA experiments using qRT-PCR analysis and two independent experiments using RNAseq. (DOCX) [file ppat.1004708.s001.docx]

Table S1.

| Target | Organism | Forward (5’-3’) | Reverse (5’-3’) |
| --- | --- | --- | --- |
| ACTB | *Bos taurus* | CTGAGCGCAAGTACTCCGTG | CAGCTAACAGTCCGCCTAGA |
| CXCL2 | *Homo sapiens* | AGGGGTTCGCCGTTCTCGGA | ATGGGGCTCAGCAGGCGGTT |
| FOS | *Homo sapiens* | GTGGGAATGAAGTTGGCACT | CTACCACTCACCCGCAGACT |
| FOS | *Bos taurus* | GCAAAACGCATGGAGTGTGT | AAAAGAGACGCAGACCCAGG |
| GAPDH | *Homo sapiens* | TCTTTTGCGTCGCCAGCCGA | ACCAGGCGCCCAATACGACC |
| HIF1A | *Homo sapiens* | GCGCGAACGACAAGAAA | GAAGTGGCAACTGATGAGCA |
| JUN | *Bos taurus* | ACGACCTTCTACGACGATGC | GCCAGATTCAGGGTCATGCT |
| JUN | *Homo sapiens* | GCCAGGTCGGCAGTATAGTC | TCTGGACACTCCCGAAACAC |
| MYC | *Homo sapiens* | TGTCAAGAGGCGAACACACA | ACCTTGGGGGCCTTTTCATT |
| RN7SL1 | *Homo sapiens* | GGAGTTCTGGGCTGTAGTGC | ATCAGCACGGGAGTTTTGAC |
| TUT1 | *Homo sapiens* | AGGCAGCCCAAGAATGGTC | GCCACCACTCAGTCCTTTCA |

Table S2.

|  | Actinomycin D shut-off | | | Metabolic labeling with 4sU | | |
| --- | --- | --- | --- | --- | --- | --- |
|  | Mock (minutes) | HCV (minutes) | Fold change | Mock (minutes) | HCV (minutes) | Fold change |
| *FOS* | 45 | 77 | 1.71 | 90 | 235 | 2.61 |
| *TUT1* | 79 | 174 | 2.20 | 91 | 177 | 1.94 |
| *MYC* | 29 | 54 | 1.86 | 207 | 343 | 1.66 |
| *JUN* | 46 | 90 | 1.96 | 137 | 267 | 1.95 |
| *VEGFA* | 67 | 128 | 1.91 | 48 | 103 | 2.17 |
| *CXCL2* | 69 | 125 | 1.81 | 95 | 142 | 1.49 |

Table S3.

|  | qRT-PCR | RNAseq |
| --- | --- | --- |
| *FOS* | 3.16 | 8.5 |
| *JUN* | 4.58 | 3.37 |
| *MYC* | 2.67 | 4.07 |
| *VEGFA* | 1.77 | 2.02 |
| *HIF1A* | 1.54 | 1.51 |
| *CXCL2* | 1.51 | 1.33 |

**SUPPLEMENTAL EXPERIMENTAL PROCEDURES**

**In vitro RNA decay assays**

Templates for *in vitro* transcriptions were constructed by inserting the following viral sequences into the pGEM-4 vector. The 5’-most 200 nt of the JEV 3’ UTR (10395-10594 nt of TC2009-1 strain GenBank: JF499790.1) predicted to harbor sfRNA-forming structures [36] was assembled by overlap extension PCR and inserted into the EcoRI and HindIII sites. The 5’ half of the DENV-2 3’ UTR was amplified from cDNA made from total RNA of cells infected with the Jamaica 1409 strain (10273-10491 nt; GenBank: M20558.1) and cloned into EcoRI and HindIII sites. The HCV 5’ UTR (1-389 nt; GenBank: M67463.1) was subcloned from HCV16LUC plasmid (Bradrick et al., [3]) into the EcoRI and Xba1 sites. The eGFP open reading frame was subcloned from peGFP-N1 (679-1398 nt, GenBank U55762.1) into the EcoRI and HindIII sites of pGEM-4. The 310 nt 3’ UTR of Sindbis virus (SINV) was inserted into the EcoRI and PstI sites of pGEMA60 described in [S11]. Finally, the BVDV 5’ UTR (1-440 nt; GenBank: DQ088995.2) and the Venezuelan Equine Encephalitis virus (VEEV) 3’ UTR (11330-11446 nt; GenBank L01443.1) were amplified from infected cell RNA and inserted into the EcoRI and HindIII sites of pGEM-4.

Internally radiolabeled, 5’ monophosphorylated RNAs were generated using SP6 polymerase as described previously [S1]. All pGEM-4 constructs containing viral UTRs were linearized with HindIII to create transcription templates. pGEM-4 control RNAs were linearized with SmlI (competitor RNAs) or EarI (reporter RNAs) to generate 390 and 168 nt RNAs, respectively. *In vitro* 5’-3’ decay assays and competitions were performed essentially as described in Moon et al. [41] using HeLa S100 cytoplasmic extracts [S2] under conditions that favor 5’-3’ decay [47] or with recombinant yeast XRN1 enzyme (rXRN1; New England Biolabs). For sfRNA formation assays, approximately 30 fmol of each reporter RNA was used. For arthropod-borne flavivirus RNA competition assays, ~30 fmol of a pGEM-4 reporter RNA was incubated with a 30-fold molar excess of the pGEM-4 control competitor, the DENV-2 3’ UTR RNA or the JEV 3’ UTR RNA. The average +/- standard deviation of the percent RNA remaining in each time point from two independent experiments is reported and t-tests indicated differences between the percent reporter RNA remaining in the presence of viral 3’ UTR competitors and the control RNA competitor. To characterize the inhibition of XRN1 by the formation of DENV-2 sfRNA, increasing amounts of rXRN1 (40, 80, or 120 fmol) were titrated into a reaction containing 1500 fmol of competitor RNA and 30 fmol of reporter RNA. At 1 minute post incubation, RNAs were separated on 5% denaturing PAGE gels and visualized by phosphorimaging. The average +/- standard deviations of the percent reporter RNA remaining for each condition from three independent experiments are reported with p-values derived by comparing the DENV-2 competitor and the Control competitor reactions by t-test. For HCV and BVDV RNA competition assays, a 66.7-fold molar excess of the pGEM-4 control competitor or the DENV-2, HCV or BVDV competitor RNAs were used. The average percent reporter RNA remaining +/- standard deviation from three independent experiments are depicted with significance assessed by t-test comparing the rate of decay of the reporter transcript in the presence of viral RNA competitors or a control competitor RNA.

**Cell culture and viral infections**

Huh7.5 cells were maintained in Dulbecco’s Modified Minimal Medium (DMEM) supplemented with 10% fetal bovine serum and nonessential amino acids. HCV (JFH-1 strain) infections of Huh7.5 cells were performed at an MOI of 0.1 for either 72 or 120 hours. Huh7.5 cells stably harboring HCV replicon RNA (I_389_/NS3-3′ described in Lohmann et al., [S3]) were maintained in supplemented DMEM with 0.5 mg/ml G418. Rates of HCV infection were determined by quantitative immunofluorescence using C750 anti-core antibody (Abcam) and a high content imager (Arrayscan). Cytopathic BVDV infections were performed in MDBK (maintained in Minimal Essential Medium with 10% horse serum) cells using an MOI of 10 and all samples were collected at 24 hpi. For transfection experiments, 293T cells were grown in DMEM with 10% fetal bovine serum.

**Transfections, northern blotting and RNase protection assays**

The HCV 5’ UTR (1-389 nt; GenBank: M67463.1) or the BVDV 5’ UTR (1-440 nt; GenBank: DQ088995.2) were subcloned into the NotI site of peGFP-N1. Equal quantities of peGFP-N1 or peGFP-N1 containing the HCV or BVDV 5’ UTR were co-transfected with an siRNA targeting the eGFP open reading frame (5’-GCAAGCUGACCCUGAAGUUCAU) into 293T cells using Lipofectamine 2000 according to the manufacturer’s protocol. Total RNA was collected at 48 hpt with TRIzol and isolated per the manufacturer’s instructions. To visualize the GFP mRNA and GFP-HCV or GFP-BVDV RNA decay intermediates, northern blotting was performed as described in Moon et al. [41] using a probe to the eGFP sequence downstream of the HCV or BVDV insert. RNase protection assays were performed using 5 μg of total RNA isolated from mock or BVDV infected MDBK cells, mock or JFH1 infected Huh7.5 cells, or human 293T cells transfected as described above with either GFP alone or GFP containing the BVDV or HCV 5’ UTR and an anti-GFP siRNA, essentially as described [S4]. Antisense RNAs to the HCV and BVDV 5’ UTRs were generated by linearizing the pGEM-4 constructs described above with EcoRI and synthesizing internally radiolabeled transcripts with T7 polymerase. Hybridized, protected RNA fragments were separated on denaturing (urea) 5% polyacrylamide gels and visualized by phosphorimaging. The depicted images for northerns and RNase protection assays are representative of at least three independent experiments.

**Assessment of mRNA abundance, synthesis and decay**

Actinomycin-D shutoffs and RT-qPCR to assess mRNA stability were performed at 72 hpi (HCV infected vs mock infected), 24 hpi (BVDV infected or mock infected) or 48 hpt (GFP vs GFP-HCV) as described in Moon et al. [41]. For analysis of mRNA stability in XRN1 knockdown cells, GFP or GFP-HCV plasmids were co-transfected with siRNA to eGFP and the control plasmid pLKO.1 or XRN1 shRNA as described in Moon et al. [41] and actinomycin D shutoffs performed 48 hpt. Relative mRNA abundances were derived from untreated cells. Gene expression analysis was performed using the ΔΔCt method, with human transcripts normalized to *GAPDH* and bovine transcripts normalized to *ACTB*. Primers are listed in Supplementary Table 1. Representative decay curves are shown, with the average +/- standard deviation of three independent infections or transfections are reported. Student’s t-test was used to determine significance.

Global analyses of mRNA synthesis, decay, and abundance were performed by metabolic labeling of mock infected or JFH1 HCV infected Huh7.5 cells at 120 hpi using 4-thio uridine as described in Dölken et al. [52]. Briefly, in duplicate biological experiments, cells were labeled for one hour prior to harvest with 4sU, then RNA was isolated using TRIzol. 4sU-labeled RNA was then covalently conjugated to Biotin-HPDP and isolated using MyOne Streptavidin C1 coated Dynabeads (Life Technologies). RNA-seq libraries were constructed and sequenced for labeled RNA, unlabeled RNA and total RNA using the Scriptseq v2 library preparation kit (Epicentre) following ribosome depletion using Ribo-Zero (Epicentre).

Sequence data was mapped using TopHat2 [S5]. Read counts per gene were then assessed using the HTseq package [S6] and normalized using the edgeR package [S7]. Normalized counts per million (cpm) per gene were filtered for abundance (>1 read per million in all samples) and total, labeled and unlabeled RNA values were then compared to determine global changes in abundance, synthesis and decay as in Dölken et al. [51]. Decay rates were calculated by the natural log of 1 minus the RNA input normalized ratio of labeled cpm over unlabeled cpm: ln[1-a*(labeled cpm)/(unlabeled cpm)]. From this decay rate we calculated an instantaneous mRNA half-life and could compare changes in transcript stability between mock and infected states. Due to library size differences, the two replicates were compared independently, and then averaged for all analyses, with high correlation of comparisons throughout. Differential expression of total mRNA abundance and labeled mRNA (transcription) was determined using edgeR [S7], then Gene Ontology analysis was performed using InnateDB [54]. Analysis of transcription factor enrichments was performed using GSEA [S8].

**Analysis of uncapped or polyadenylated and capped mRNAs**

Total RNA from naïve Huh7.5 cells, HCV infections or Huh7.5 cells harboring a replicon HCV construct was fractionated into capped and uncapped pools using an antibody that recognizes the methyl guanosine cap structure (Synaptic Systems) as described in Moon et al. [41]. The average +/- standard deviation of the relative abundance of *TUT1* and *FOS* in the uncapped fraction relative to the 10% input derived from RNAs of three independent infections is reported. For analysis of capped and polyadenylated mRNA abundances, the capped (bound) fraction of mRNAs was subjected to reverse transcription (Improm-II; Promega) using oligod(T) primers to selectively amplify polyadenylated transcripts by qPCR using *GAPDH* as a reference gene. The reported values are the average +/- standard deviation of three independent experiments from mock infected and HCV infected Huh7.5 cells. Student’s t-test was done to assess significance.

**Western blotting**

Cell lysates were obtained from MDBK cells either mock infected or infected with cytopathic BVDV (MOI of 10) at 24 hpi. Western blotting was performed using antibodies to GAPDH (6C5, Chemicon), FOS (H-125, Santa Cruz Biotechnology), JUN (H-79, Santa Cruz Biotechnology), TUBA1A (B-5-1-2, Sigma Aldrich) or XRN1 (C-1, Santa Cruz Biotechnology) as described in Barnhart et al. [60]. Antibodies to Xrn1 and RPL19 (both from (Santa Cruz Biotechnology) were used for quantitative Western blotting using an Odyssey infrared scanner (LI-COR Biosciences). The average +/- standard deviation of FOS and JUN abundance relative to GAPDH from three independent infections is reported with p-values obtained using Student’s t-test.

**Cloning of XRN1-mediated decay intermediates**

*In vitro* transcriptions were performed using the MEGAscript® SP6 kit (Ambion) with pGEM-4 containing the HCV or BVDV 5’ UTR linearized with HindIII and a 10-fold excess of GMP to create monophosphorylated transcripts. Following gel purification to remove excess unincorporated nucleotides, 2.5 µg of each RNA was incubated with recombinant yeast XRN1 (New England Biolabs) for 0 minutes, 5 minutes (BVDV RNA) or 15 minutes (HCV RNA) at 37^o^C. Reactions were stopped by addition of 400 μL HSCB (25 mM Tris-HCl pH 7.6, 400 mM NaCl, 0.1% sodium dodecyl sulfate) followed by phenol-chloroform extraction and ethanol precipitation. Remaining RNA was then separated on a 5% denaturing polyacrylamide gel and stained for 30 minutes with SYBR green I (Invitrogen). Indicated bands (S1 Fig) were purified from the gel and circularized using T4 RNA ligase. Ligation products were reverse transcribed with Improm-II (Promega) using a specific reverse primer (listed below) to the BVDV or HCV RNA. Products were then amplified using Go Taq® Flexi DNA polymerase (Promega) and cloned into the pGEM T-Easy vector (Promega) for transformations of DH5α cells. Colony screens were performed by PCR with GoTaq® Flexi DNA polymerase (Promega) using primers specific to the HCV or BVDV RNA (listed below) to amplify the junction between the 3’ and 5’ RNA ends. PCR products were then gel purified and six clones per decay intermediate were sequenced. The resulting mapped 5’ ends of the sequenced RNAs were aligned to the HCV or BVDV 5’ UTRs. Primers used for these analyses were: HCV Fw: 5-GCGAAAGGCCTTGTGGTACTGCCTGATAG; HCV Rv: 5’-GGTTTATCCAAGAAAGGACCCGGTCGTC; BVDV Fw: 5’-aggcccactgttctgctactaaaaatctct; BVDV Rv: 5’-cagtcggttagaactgcttttacctgggcg.

**Supplemental References**

S1. Wilusz, J., and Shenk, T. (1988) [A 64 kd nuclear protein binds to RNA segments that include the AAUAAA polyadenylation motif.](http://www.ncbi.nlm.nih.gov/pubmed/2830023) Cell *52,* 221-228.

S2. Ford, LP, Wilusz, J. (1999) [An in vitro system using HeLa cytoplasmic extracts that reproduces regulated mRNA stability.](http://www.ncbi.nlm.nih.gov/pubmed/10075879) Methods 17: 21-27.

S3. Lohmann, V, Körner, F, Koch, J, Herian, U, Theilmann, L, Bartenschlager, R. (1999) [Replication of subgenomic hepatitis C virus RNAs in a hepatoma cell line.](http://www.ncbi.nlm.nih.gov/pubmed/10390360) Science 285: 110-113.

S4. Rio, DC, Ares, M, Hannon GJ, Nilsen TW. (2011) RNA a Laboratory manual. Cold Spring Harbor Laboratory Press. Cold Spring Harbor, New York.

S5. Kim, D, Pertea, G, Trapnell, C, Pimentel, H, Kelley, R, Salzberg, SL. (2013) [TopHat2: accurate alignment of transcriptomes in the presence of insertions, deletions and gene fusions.](http://www.ncbi.nlm.nih.gov/pubmed/23618408) Genome Biol 14:R36.

S6. Anders, S, Pyl, PT, Huber, W. (2014) HTSeq-A python framework to work with high-throughput sequencing data. BioRxiv, doi: http://dx.doi.org/10.1101/002824.

S7. Robinson, MD, McCarthy, DJ, Smyth, GK. (2009) [edgeR: a Bioconductor package for differential expression analysis of digital gene expression data.](http://www.ncbi.nlm.nih.gov/pubmed/19910308) Bioinformatics 26: 139-140.

S8. Subramanian, A, Kuehn, H, Gould, J, Tamayo, P, Mesirov, JP. (2007) [GSEA-P: A desktop application for Gene Set Enrichment Analysis](http://bioinformatics.oxfordjournals.org/cgi/reprint/btm369?ijkey=ksH1dJrTP7J4T6D&keytype=ref). Bioinformatics 1: 3251-3253.

S9. Sievers, F, Wilm, A, Dineen, DG, Gibson, TJ, Karplus, K, et al. (2011) Fast, scalable generation of high-quality protein multiple sequence alignments using Clustal Omega. Mol Syst Biol 7: 539.

S10. Goujon, M, McWilliam, H, Li, W, Valentin, F, Squizzato, S, et al. (2010) A new bioinformatics analysis tools framework at EMBL-EBI. 2010. Nucleic Acids Res 38: W695-699.

S11. Garneau, NL, Sokoloski, KJ, Opyrcha,l M, Neff, CP, Wilusz, CJ, Wilusz, J (2008) [The 3' untranslated region of sindbis virus represses deadenylation of viral transcripts in mosquito and mammalian cells.](http://www.ncbi.nlm.nih.gov/pubmed/17977976) J Virol 82:880-892.
