# Supplementary figures and images for: XRN1 Stalling in the 5’ UTR of Hepatitis C Virus and Bovine Viral Diarrhea Virus Is Associated with Dysregulated Host mRNA Stability
Source: PLoS Pathog. 2015 Mar 6;11(3):e1004708. doi: 10.1371/journal.ppat.1004708 (PMC4352041; doi:10.1371/journal.ppat.1004708)

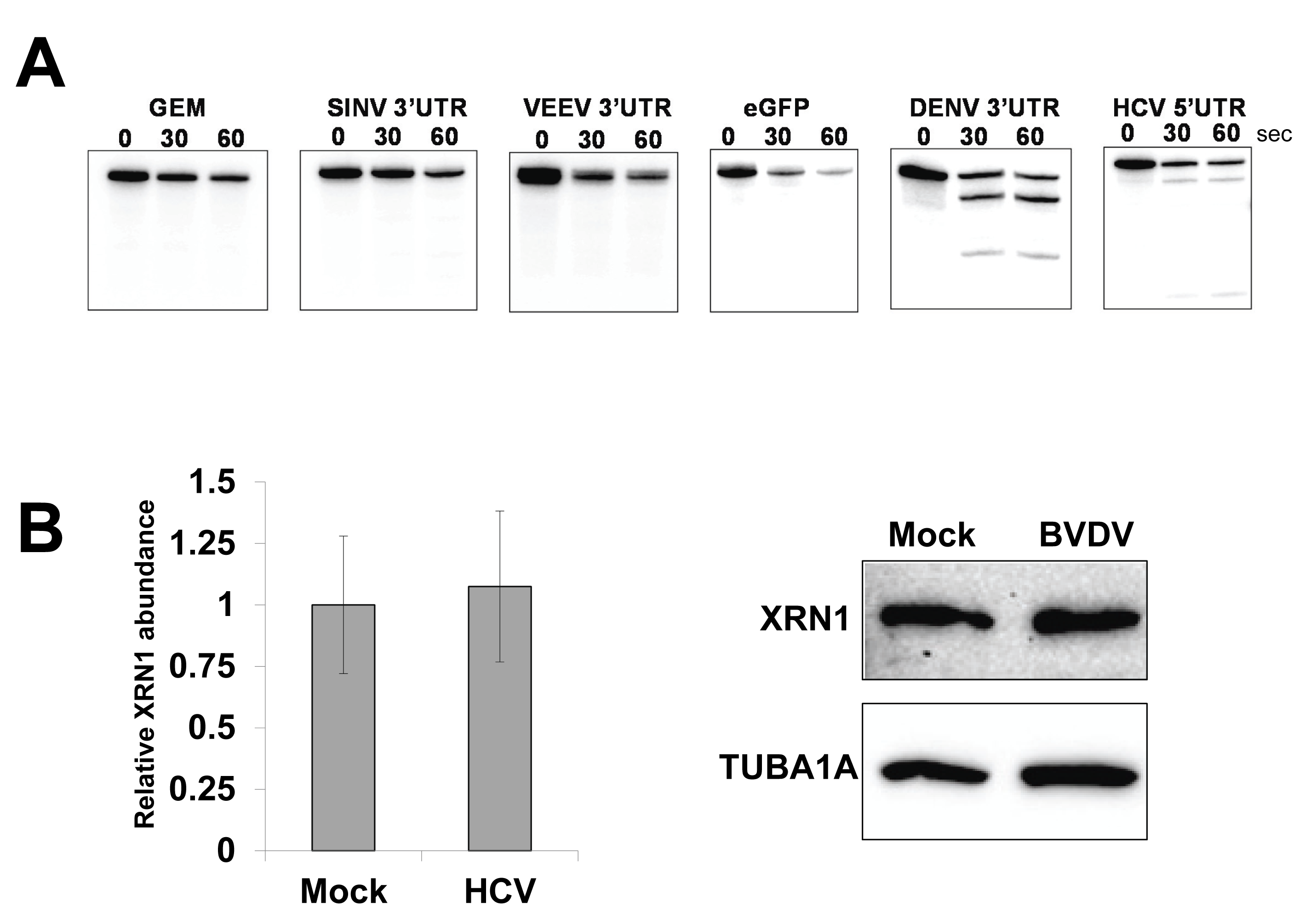

Supplement: S1 Fig — Related to Figs. 1 and 4. Panels A. A series of radiolabeled RNAs containing a 5’ monophosphate was incubated with recombinant XRN1 for 0, 30 or 60 seconds. Reaction products were analyzed on a 5% denaturing acrylamide gel. Panel B. XRN1 levels in Huh7.5 cells (left panel) or MDBK cells (right panel) were analyzed by western blotting during mock or either HCV (left) or BVDV (right) infection. Average XRN1 levels from three HCV infections +/− standard deviation relative to RPL19 control are shown. Tubulin (TUBA1A) was used as a loading control in the gel on the right (representative of blots from three independent infections). (TIF) [file ppat.1004708.s002.tif]

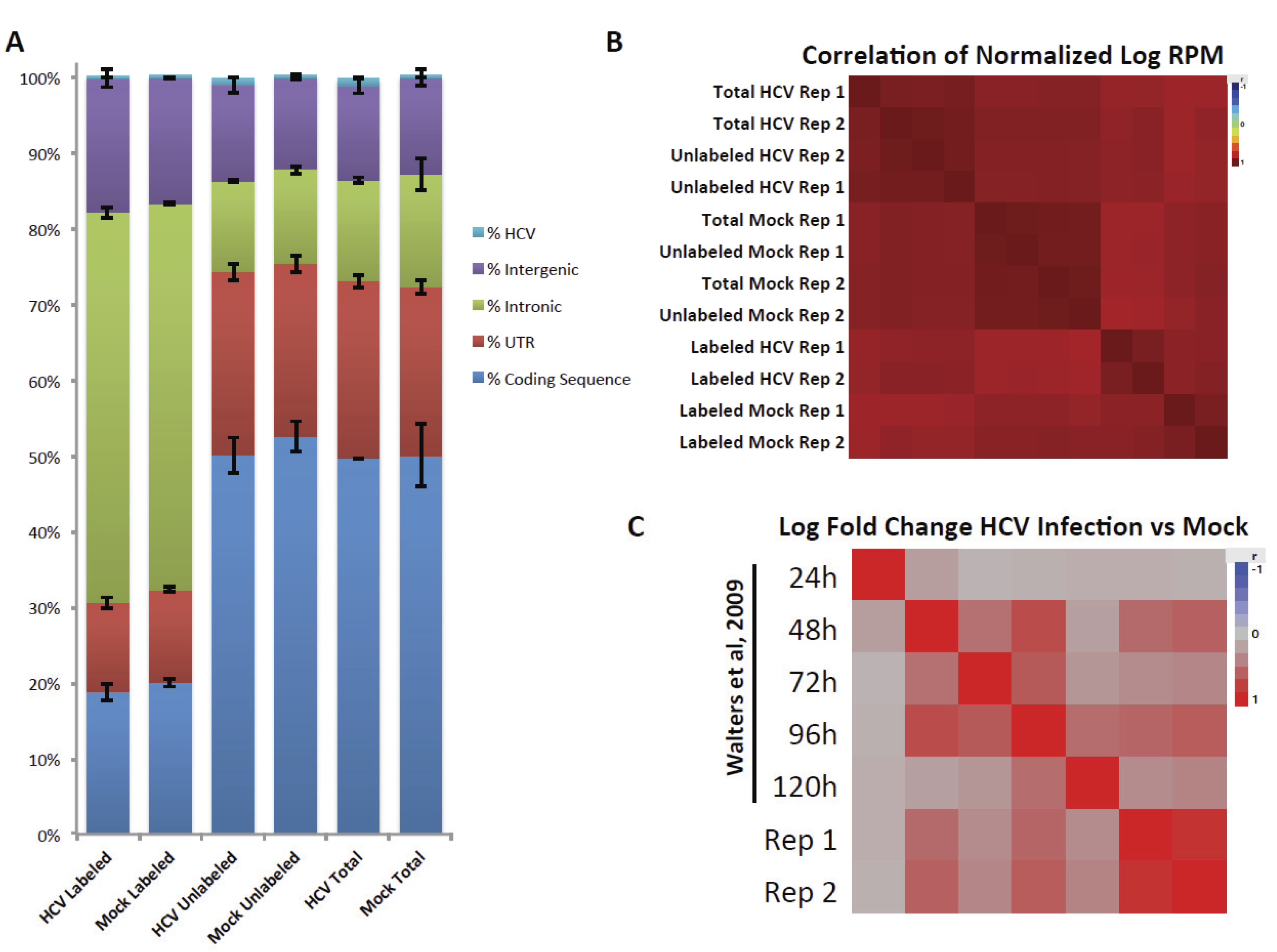

Supplement: S2 Fig — Related to Fig. 5. Panel A. HCV and Mock infected Huh7.5 cells were subjected to 4sU labeling for one hour prior to harvest, then RNA was separated into 4sU labeled, unlabeled and total populations and subject to Illumina sequencing. Tophat2 mapped reads were categorized as intronic, coding sequence, intergenic, UTR or HCV using PicardTools CollectRNASeqMetrics function. Reads mapping to the HCV genome in infected samples accounted for ∼1% of all mapped reads. Error bars represent standard deviation. Panel B. Normalized log2 transformed sequencing data correlated highly and samples clustered together. Panel C. Changes in mRNA abundance correlate well with previously published HCV infections. A total of 612 differentially expressed mRNAs from Walters et al. [43] at time points from 24–120 hours were compared to mRNA abundance changes observed in Mock versus HCV infected samples at 120 hours. (TIF) [file ppat.1004708.s003.tif]

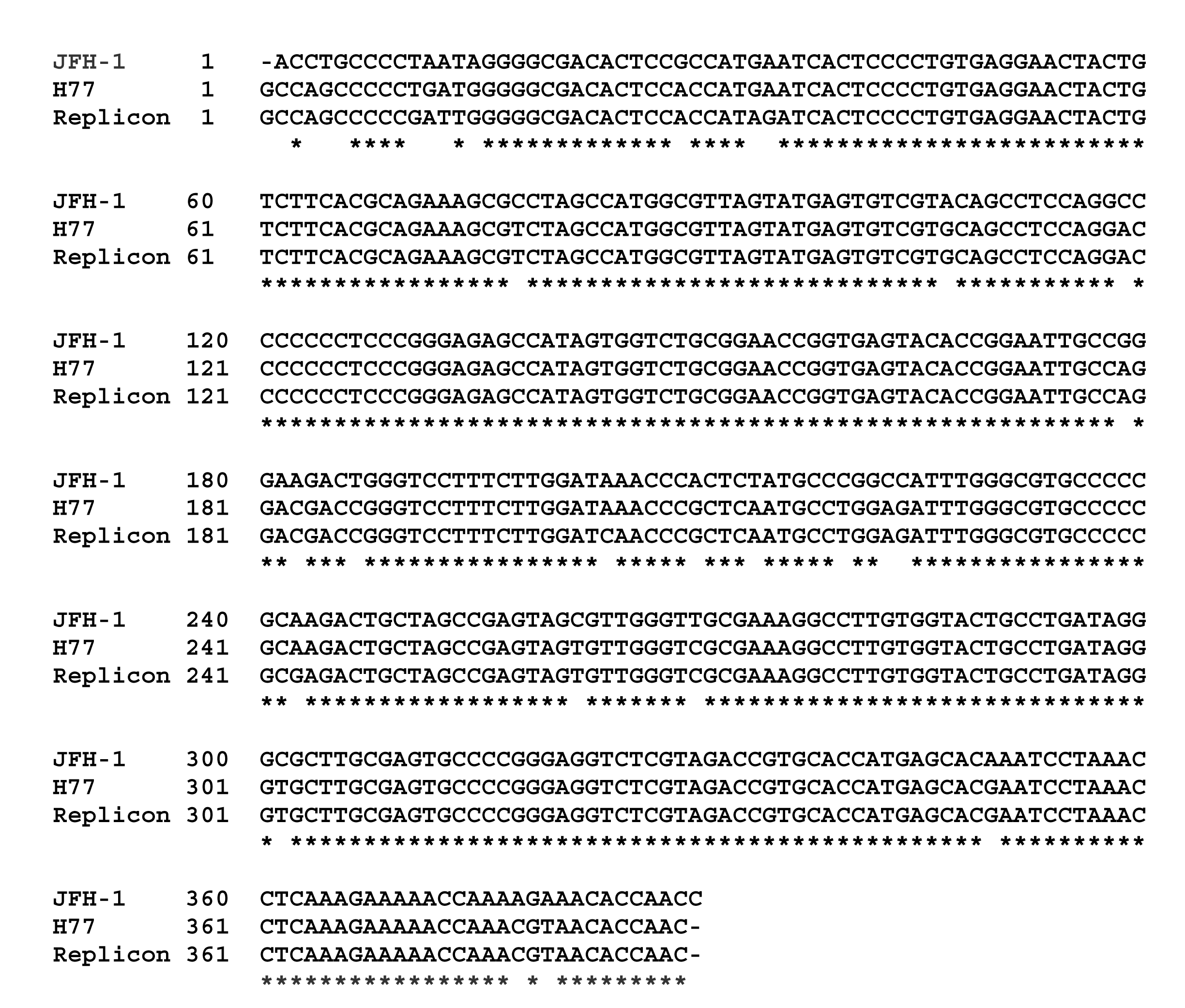

Supplement: S3 Fig — Related to Figs. 1–6. Clustal Omega alignment [S9, S10] of the sequences of the 5’ UTRs of the three strains of HCV used in the study. JFH-1 is the wild-type HCV infectious virus used in Figs. 2D, 3A, 4, and 5. The H77 5’ UTR was cloned into pGEM-4 and peGFP-N1 vectors for Figs. 1, 2, and 3; and the Replicon was used for Fig. 2D. Note that the various 5’ UTRs are over 92.5% similar to each other (determined by the clustal 2.1 percent identity matrix). (TIF) [file ppat.1004708.s004.tif]

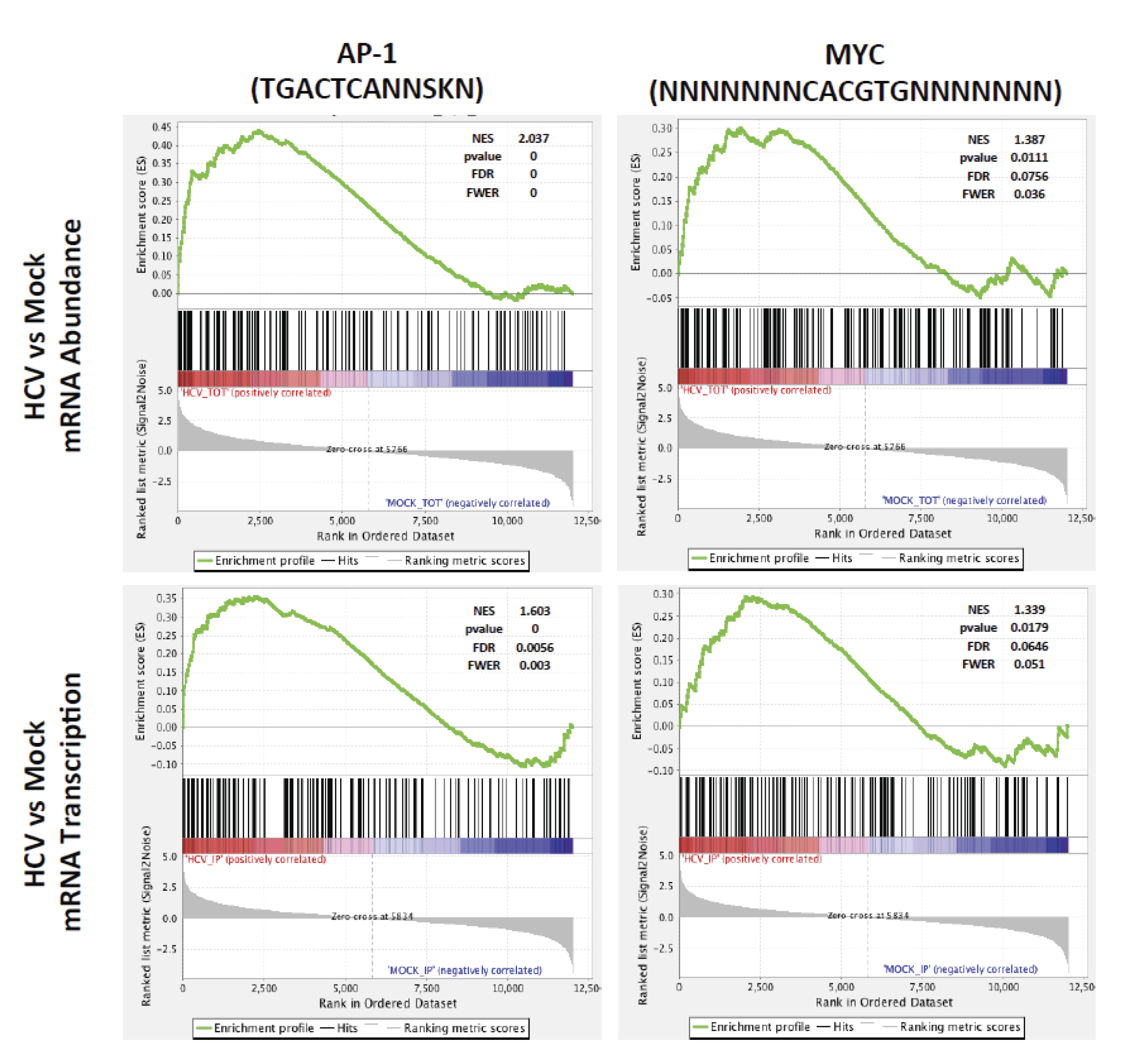

Supplement: S4 Fig — Related to Figs. 5–7. MSigDB datasets defining mRNAs with binding sites matching a set of predicted binding motifs for the FOS and JUN heterodimer AP-1 (left panels) and MYC (right panels) were evaluated for correlation with rank ordered changes in mRNA abundance (top panels) and mRNA transcription rate (bottom panels). (TIF) [file ppat.1004708.s005.tif]
